# Beyond genome-wide association studies: Investigating the role of noncoding regulatory elements in primary sclerosing cholangitis

**DOI:** 10.1097/HC9.0000000000000242

**Published:** 2023-09-27

**Authors:** Henry E. Pratt, Tong Wu, Shaimae Elhajjajy, Jeffrey Zhou, Kate Fitzgerald, Tom Fazzio, Zhiping Weng, Daniel S. Pratt

**Affiliations:** 1 Program in Bioinformatics and Integrative Biology, Department of Biochemistry and Molecular Biotechnology, University of Massachusetts Medical School, Worcester, Massachusetts, USA; 2 Department of Molecular, Cell, and Cancer Biology, University of Massachusetts Chan Medical School, Worcester, Massachusetts, USA; 3 Program in Innate Immunity, Department of Medicine, University of Massachusetts Chan Medical, School, Worcester, Massachusetts, USA; 4 Autoimmune & Cholestatic Liver Center, GI Division, Massachusetts General Hospital, Boston, Massachusetts, USA

## Abstract

**Background:**

Genome-wide association studies (GWAS) have identified 30 risk loci for primary sclerosing cholangitis (PSC). Variants within these loci are found predominantly in noncoding regions of DNA making their mechanisms of conferring risk hard to define. Epigenomic studies have shown noncoding variants broadly impact regulatory element activity. The possible association of noncoding PSC variants with regulatory element activity has not been studied. We aimed to (1) determine if the noncoding risk variants in PSC impact regulatory element function and (2) if so, assess the role these regulatory elements have in explaining the genetic risk for PSC.

**Methods:**

Available epigenomic datasets were integrated to build a comprehensive atlas of cell type–specific regulatory elements, emphasizing PSC-relevant cell types. RNA-seq and ATAC-seq were performed on peripheral CD4^+^ T cells from 10 PSC patients and 11 healthy controls. Computational techniques were used to (1) study the enrichment of PSC-risk variants within regulatory elements, (2) correlate risk genotype with differences in regulatory element activity, and (3) identify regulatory elements differentially active and genes differentially expressed between PSC patients and controls.

**Results:**

Noncoding PSC-risk variants are strongly enriched within immune-specific enhancers, particularly ones involved in T-cell response to antigenic stimulation. In total, 250 genes and >10,000 regulatory elements were identified that are differentially active between patients and controls.

**Conclusions:**

Mechanistic effects are proposed for variants at 6 PSC-risk loci where genotype was linked with differential T-cell regulatory element activity. Regulatory elements are shown to play a key role in PSC pathophysiology.

## Graphical abstract

**Figure:**
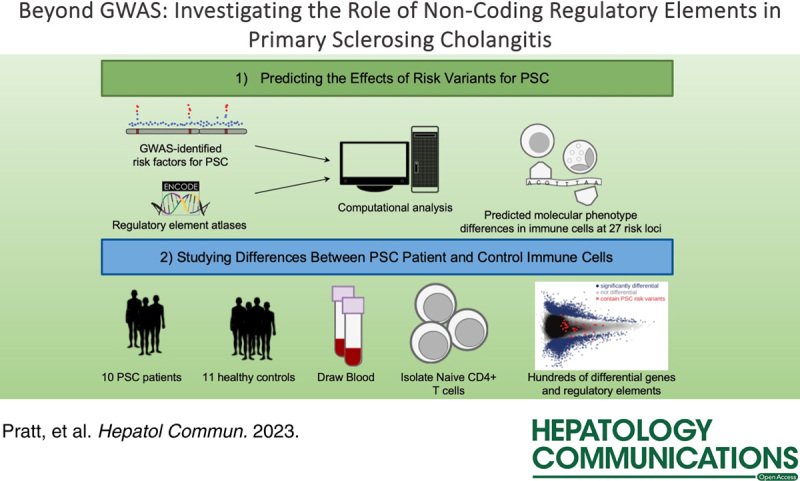

## INTRODUCTION

Primary sclerosing cholangitis (PSC) is a rare immune-mediated liver disorder marked by progressive structuring of the biliary tree. Many patients progress to end-stage liver disease.^1^ The pathophysiology of PSC is poorly understood. Hypotheses include aberrant lymphocyte homing to the biliary tract,^2,3^ leakage of antigens into portal circulation,^4^ and toxic damage from bile acids.^5,6^ Heterogeneity in clinical features further complicates investigation of this complex disease. Comorbid inflammatory bowel disease (IBD) occurs in ∼70% of PSC patients, typically resembling ulcerative colitis (UC),^1^ although clinical and genetic features suggest PSC/IBD may be a distinct disease entity.^7^ There is no proven medical therapy for PSC, although there is heterogeneity in individual response to therapies including ursodeoxycholic acid^6^ and oral antibiotics including vancomycin.

There is a clear genetic risk for the development of PSC, with a hazard ratio of ∼11 for first-degree relatives.^8^ Genome-wide association studies (GWAS) have associated thousands of single nucleotide polymorphisms (SNPs) with complex traits and diseases. However, linking these SNPs to pathophysiological insights is immensely challenging as most disease-associated SNPs reside within 98.5% of the genome that does not code for proteins.^9^ Several PSC GWAS^7,10,11^ have been performed, associating 30 genomic loci with PSC; however, most of these are noncoding, and their pathophysiological role remains opaque.

Noncoding DNA contains regulatory elements, including promoters, enhancers, insulators, and repressors/silencers, which restructure chromatin and recruit transcription factors (TFs) to modulate gene expression. Several consortia, including the Encyclopedia of DNA Elements (ENCODE) Project, have aimed to catalog regulatory elements genome-wide.^12^ Regulatory elements, particularly enhancers, are only active in certain cell types: some enhancers only become active during terminal differentiation of a cell lineage [eg, regulatory T cells (Tregs)] or in response to stimuli.^13,14^ Autoimmune-related GWAS SNPs are enriched within leukocyte-specific enhancers,^15^ and thus might alter the fraction of patient cells belonging to different lineages or how patient immune cells respond to stimuli. Understanding these roles will only be possible through a high-resolution, comprehensive map of lineage-specific enhancers in a sufficiently broad panel of immune cell lineages and states.

Here, we undertake a 2-phase pilot study of the role of noncoding regulatory elements in the PSC pathogenesis. First, we perform computational analysis to prioritize PSC-risk variants likely to be gene regulatory. Many studies^13,14,16–21^ have profiled the epigenetic landscapes of immune cells and other pathophysiologically relevant cell types such as cholangiocytes and colonic epithelium. Public datasets are invaluable: collecting tissue samples from living donors poses risk, and with public datasets, we can form hypotheses about risk SNPs so experiments in patients and healthy controls can be targeted to relevant cell types. We integrate 293 public ATAC-seq datasets to assemble a comprehensive map of regulatory elements then use this map to identify cell types where heritable factors for PSC likely influence regulatory element activity.

Second, on the basis of these computational results, we profile the chromatin and transcriptomic landscapes of peripheral CD4^+^ T cells in 10 PSC patients and 11 healthy controls—a sufficient dataset for identifying allele-specific effects at PSC-implicated regulatory elements. Our results illustrate an essential role for immune regulatory elements in PSC, suggest pathophysiological roles for several distinct immune cell types and states, and prioritize novel candidate risk genes for follow-up study.

## METHODS

### Definition of PSC-risk loci

We obtained PSC-risk variants from the EBI catalog (https://www.ebi.ac.uk/gwas/studies/GCST006670).^47^ SNPs from the HLA locus were filtered using bedtools. Risk SNPs in the linkage disequilibrium (LD) set are those with *r* ^2^ > 0.7 in the European population using LD information from 1000 Genomes. The fine-mapped set was defined with FINEMAP using 1000 Genomes European LD information assuming one causal SNP per locus with default parameters otherwise; credible SNPs were defined as any SNP in the FINEMAP 95% credible set with an individual posterior probability >1% of being causal.

### Deleterious coding annotations

We annotated PSC-risk SNPs from the LD and FINEMAP steps with deleterious coding annotations using SIFT.^25^ We performed annotations using the offline command-line version and version 83 of the hg38 annotations database obtained from: https://sift.bii.a-star.edu.sg/sift4g/public//Homo_sapiens/GRCh38.83.chr.zip.

### Differential ATAC-seq analysis

Differential analysis was performed using csaw with loess normalization.^36^ For each comparison set, we identified active ENCODE rDHSs to test by selecting elements meeting the *Z* > 1.64 threshold in at least 3 of the datasets across both conditions. We obtained read counts at each rDHS from the filtered BAM alignment files produced by Picard and input these to csaw using a custom Python script available at: https://www.github.com/weng-lab/PSC-analysis/.

We used bedtools to produce the sets shown in Figure 3D. We separately took the union of all T-cell receptor (TCR)-responsive elements in the 5 CD4^+^ T-cell lineages analyzed and the 4 CD8^+^ T-cell lineages analyzed. We then used the bedtools intersect command to identify elements shared between the sets and unique to the sets. BED files for the sets are available in the code repository at: https://www.github.com/weng-lab/PSC-analysis/.

### Partitioned LD score regression

Heritability enrichment for PSC was computed on various partitions of regulatory elements using partitioned LD score regression^48^ with the baseline v2.2 model. Conditional *p* values were computed against the baseline model using the –print-coefficients flag. Code is available at: https://github.com/weng-lab/ldr. PSC summary statistics were obtained from: https://www.ebi.ac.uk/gwas/studies/GCST006670. Summary statistics for UC, atrial fibrillation, and educational attainment are available at: https://www.github.com/weng-lab/PSC-analysis/.

For bulk embryonic tissue and immune cell ATAC-seq data, enrichment was computed individually for each dataset using all active regulatory elements with *Z* >1.64. For single-cell data, enrichment was computed for the top 100,000 most active rDHSs in each cell type based on pseudo-bulk signal profiles.

### Allele-specific analysis

For each patient and control in our dataset, we extracted reads containing each SNP using samtools, then filtered reads for reference bias using the WASP framework.^49^ Individuals having at least 5 reads for an SNP were considered heterozygotes if both alleles were observed at least once and homozygotes if only 1 allele was observed in the filtered reads. Individuals with <5 reads containing a given SNP were excluded.

At each SNP, we summed the total number of reads for each allele across all heterozygotes to obtain a reference and alternative allele fraction. For each of the 3 datasets, patient versus control, unstimulated T cells, and stimulated T cells, we fit a separate beta-binomial distribution for all the reference fractions, to account for overdispersion.^50^ We then used the beta-binomial distribution parameters for each set to test each SNP for significant allele-specific chromatin accessibility.

## RESULTS

### Fewer than 10% of PSC-associated variants are coding

**FIGURE 1:**
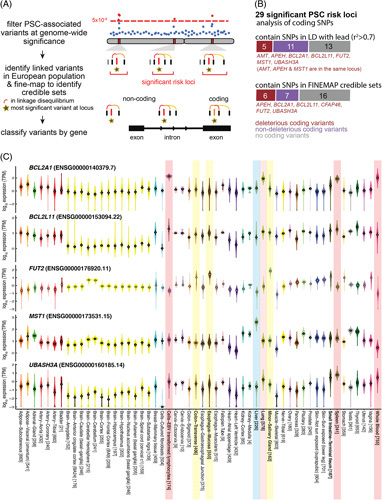
Overview of PSC-risk loci and their protein-coding genes. A, Schematic illustrating how the 29 non-HLA PSC-risk loci and credible sets of causal variants were defined. Lead variants were identified by GWAS significance; these were supplemented with fine-mapped SNPs and SNPs in LD. Finally, each locus was classified as candidate coding or noncoding based on intersection with annotated coding exons. B, Most PSC-risk loci are unlikely to cause deleterious impacts on protein-coding genes. Two horizontal stacked bar plots illustrate the numbers of loci that contain deleterious coding variants (maroon), nondeleterious coding variants (purple), and only noncoding variants (gray), according to high-LD SNPs (top) or fine-mapped SNP credible sets (bottom). Genes with deleterious coding variants are provided. C, Expression profiles for 5 genes with candidate coding variants are displayed based on GTEx RNA-seq data, highlighting immune-specific expression for *BCL2A1*, *BCL2L11*, and *UBASH3A*, and GI-specific expression for *FUT2* and *MST1*. Relevant tissues are shaded—immune (red), GI (yellow), liver (cyan), and lung (pink). Abbreviations: *BCL2L11*, Bcl2-like 11 apoptosis facilitator; GI, gastrointestinal; GWAS, genome-wide association studies; HLA, human leukocyte antigen; LD, linkage disequilibrium; *MST1*, macrophage stimulatory protein; PSC, primary sclerosing cholangitis; SNP, single nucleotide polymorphism.

We first aimed to identify candidate causal variants at the 30 genomic regions associated with PSC based on the latest GWAS^7^ (Supplemental Table S1A, http://links.lww.com/HC9/A494). The strongest association is the human leukocyte antigen (HLA) region, but its gene density and polymorphism pose unique analytical challenges when seeking causal variants.^22^ Indeed, it has been suggested that studies of multiethnic or admixed populations may be necessary to identify causal variants driving PSC-HLA associations.^23^ Therefore, here we focus on characterizing the 29 remaining non–HLA-risk loci. We used 2 complementary approaches to build credible sets of candidate causal variants at each risk locus for further analysis (Figure 1A). First, we consider all variants in strong LD (*r* ^2^>0.7) with the most significant GWAS-SNP at the locus in the European population. Second, we performed fine mapping with FINEMAP,^24^ which uses Bayesian analysis to assign a posterior probability that each SNP is a causal SNP for PSC based on European LD structure (Supplemental Methods, http://links.lww.com/HC9/A496).

These approaches identified 1064 and 377 PSC-risk variants (Supplemental Table S1B, C, http://links.lww.com/HC9/A494), of which 105 (9.9%) and 52 (13.8%) lie within coding exons. We used SIFT^25^ to predict which of these are deleterious versus tolerated coding variants. SIFT analysis was reported in the most recent PSC GWAS^7^; we extended that analysis to additional credible variants by relaxing LD stringency and including FINEMAP results. SIFT predicted deleterious effects at only 6 of the 16 PSC-risk loci containing coding variants (Figure 1B; Supplemental Table S1D, E, http://links.lww.com/HC9/A494). We reproduced previously reported annotations of deleterious mutations in *MST1* (macrophage stimulatory protein) and *BCL2L11* (Bcl2-like 11 apoptosis facilitator)^7,26,27^ and identified novel deleterious coding variants in genes at 4 additional loci: *FUT2*, *UBASH3A*, *BCL2A1*, and *CFAP46*. At the *MST1* locus, we also identified deleterious variants in *APEH* and *AMT*.

We next used RNA-seq data from GTEx^28^ to determine if these genes are expressed in pathophysiologically relevant tissues. Three are specifically expressed in lymphocytes: *BCL2A1* and *BCL2L11* are *BCL-2* family members involved in apoptosis with roles regulating immune cell proliferation and survival^29,30^; *UBASH3A* is a T-cell ubiquitin ligand family member which promotes accumulation of activated TCRs^31^ (Figure 1C). Two of the remaining genes are gastrointestinal (GI)-specific: *FUT2* catalyzes cell-surface glycan assembly, which mediates microbiome interactions,^32^ while *MST1* is a serine/threonine kinase regulating both hepatocyte quiescence and immune cell activation^33^ (Figure 1C). *CFAP46* neither GI-specific nor immune-specific, although it is involved in cilia assembly and could impact cholangiocyte function. *APEH* and *AMT* are not tissue-specific but are at the same locus as *MST1*; given its expression pattern, *MST1* seems the most likely gene association at that locus, although follow-up experimentation will be needed.

### Noncoding PSC-associated variants are strongly enriched in T-cell regulatory elements

If the remaining 23 PSC-risk loci are truly functional, we hypothesize they confer risk by noncoding effects (the primary alternative being disruption of unannotated exons). Noncoding risk variants might regulate gene expression, alter mRNA splicing, or impact binding of RNA-binding proteins. GWAS variants are well known to be enriched within regulatory elements active in physiologically relevant cell types.^12^ We primarily aimed to determine whether this holds for PSC. We built 4 panels of regulatory elements for analysis, each covering tens of cell and tissue types (Supplemental Methods, http://links.lww.com/HC9/A496). Overall, 27 of the 29 risk loci have at least 1 candidate regulatory element when considering both the LD and FINEMAP credible sets (Supplemental Table S1F, http://links.lww.com/HC9/A494).

**FIGURE 2:**
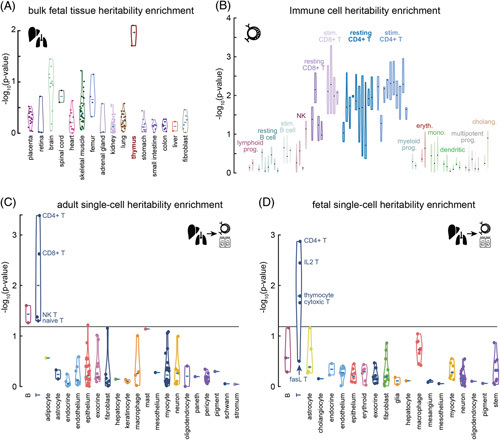
Heritability enrichment based on partitioned LD score regression for PSC-risk variants within cell type–specific regulatory elements. A, Enrichment within regulatory elements identified by DNase-seq in bulk tissues from an ENCODE embryonic panel; the thymus shows striking heritability enrichment while other tissues are not enriched or are only modestly enriched. B, Enrichment within regulatory elements active in various immune cell lineages as well as cholangiocytes based on public ATAC-seq data. T cells show the strongest enrichment. Notably, we did not observe enrichment in cholangiocyte regulatory elements. C and D, Heritability enrichment within regulatory elements from tissue-resident cell types identified using single-cell ATAC-seq data.^34^ For both fetal and adult tissues, we note enrichment only within tissue-resident lymphocytes and T cells, with a notable absence of enrichment in GI epithelial cells. Abbreviations: cholang, cholangiocytes; ENCODE, Encyclopedia of DNA Elements; eryth, erythrocytes; GI, gastrointestinal; LD, linkage disequilibrium; mono, monocytes; prog, progenitor; PSC, primary sclerosing cholangitis; stim, stimulated.

First, we assessed PSC heritability enrichment within regulatory elements identified using ENCODE data in fetal tissue homogenates.^12^ Of the 16 tissues investigated, the thymus, the site of T-cell maturation, exhibits significant enrichment (Figure 2A; Supplemental Table S2A, http://links.lww.com/HC9/A495). We next analyzed 293 public ATAC-seq datasets (Supplemental Table S2B, http://links.lww.com/HC9/A495) covering 62 FACS-sorted hematopoietic cell lineages and primary cholangiocytes. Heritability for PSC-associated variants is enriched within the lymphocyte samples, particularly antigen-stimulated T cells and resting CD4^+^ T cells. Notably, there is no significant enrichment within cholangiocyte-active elements and other hematopoietic cell types not relevant to PSC pathophysiology, such as erythroid cells (Figure 2B; Supplemental Table S2B, http://links.lww.com/HC9/A495).

We next analyzed a human single-cell ATAC-seq atlas derived from fetal and adult tissues^34^ to resolve heritability enrichment in tissue-resident cell lineages. The most recent PSC GWAS^7^ was analyzed by the atlas’ authors^34^; we refined their analysis by (i) using higher resolution regulatory elements and (ii) conditioning enrichments on a significantly expanded baseline (v2.2) model, ensuring consistency with our analysis of the other panels. We observed significant enrichment only in tissue-resident lymphocytes, particularly CD4^+^ T cells (Figure 2C, D; Supplemental Table S2C, D, http://links.lww.com/HC9/A495).^34^ There was a conspicuous absence of enrichment for colonic epithelium, cholangiocytes, hepatocytes, endothelial cells, and Paneth cells, intestinal cells producing antimicrobial peptides that could influence the microbiome (Figure 2C, D). For comparison, we also analyzed GWAS for UC, atrial fibrillation, and educational attainment. In contrast to PSC, we find significant enrichment for colonic epithelial cells, macrophages, and lymphocytes in UC, consistent with previous reports.^34,35^ Importantly, lymphocytes are not enriched for atrial fibrillation or educational attainment, highlighting that immune enrichment is specific to immune-mediated diseases. The most enriched cell types for those traits are myocytes and neurons, respectively (Supplemental Figure S1, http://links.lww.com/HC9/A496, Supplemental Table S3A, B, http://links.lww.com/HC9/A497).

### PSC-associated T-cell regulatory elements are responsive to TCR stimulation

**FIGURE 3:**
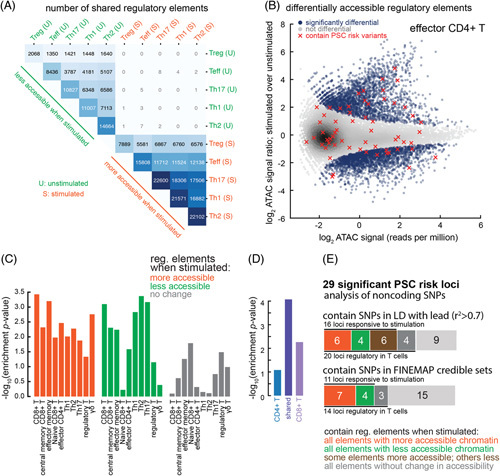
Heritability enrichment for PSC variants within TCR responsive regulatory elements. A, Heatmap displaying the number of regulatory elements whose chromatin becomes less accessible (green) or more accessible (orange) in 5 CD4^+^ T-cell lineages following TCR stimulation.^14^ The 5 T-cell lineages share many regulatory elements and the direction of their responsiveness to stimulation is nearly always preserved. B, MA plot illustrating TCR-responsive regulatory elements in effector CD4^+^ T cells (the Teff lineage in A). Regulatory elements containing PSC-risk variants are labeled as red X’s. C, Heritability enrichment for PSC variants within T-cell regulatory elements whose chromatin became significantly more accessible (orange), less accessible (green), or unchanged (gray) following TCR stimulation. D, Heritability enrichment within TCR-responsive elements unique to CD4^+^ T cells (left bar), shared between CD4^+^ and CD8^+^ T cells (center bar), and unique to CD8^+^ T cells (right bar). Enrichment is most prominent in the shared set, although some enrichment is observed for the set unique to CD8^+^ T cells too. E, The number of PSC-risk loci containing variants that impact 1 or more T-cell regulatory elements based on LD (top stacked bar) or fine-mapped credible variants (bottom stacked bar). Orange, the locus contains one or more TCR-upregulated elements; green, the locus contains one or more TCR-downregulated elements; brown, the locus contains elements TCR-responsive in both directions; dark gray, the locus contains T-cell elements but none responsive to TCR stimulation; light gray, the locus contains no T-cell regulatory elements. Abbreviations: LD, linkage disequilibrium; MA, Log Ratio vs Mean Average Plot; PSC, primary sclerosing cholangitis; TCR, T-cell receptor.

Following TCR stimulation, tens of thousands of regulatory elements become more or less chromatin-accessible to regulate downstream target genes in the signaling cascades mediating the cellular response. The datasets we analyzed include ATAC-seq on 5 TCR-stimulated CD4^+^ T-cell lineages: Tregs, Th1 cells, Th2 cells, Th17 cells, and effector T cells.^14^ We used our high-resolution regulatory elements and the csaw tool with loess normalization^36^ to identify regulatory elements differentially accessible prestimulation and poststimulation. In agreement with previous results,^14^ ∼30,000 elements increase accessibility following TCR stimulation (Figure 3A). Our methodology enabled us to identify ~10,000 regulatory elements which decrease accessibility following stimulation, a greater number than identified previously.^14^ Differential elements are largely shared across the 5 lineages, and their directionality is nearly perfectly conserved—only 3 elements become more accessible poststimulation in one lineage but less accessible in another (Figure 3A). Each lineage uses ∼60,000–120,000 regulatory elements, so most elements in each lineage are not TCR-responsive.

We used partitioned LD score regression to assess heritability enrichment for PSC within TCR-responsive elements. Forty PSC-risk variants overlap TCR-responsive elements, of which 16 are shared between the LD and FINEMAP credible sets and 24 are unique to the LD set (Figure 3B); this represents significant enrichment compared with random permutations (Supplemental Methods, http://links.lww.com/HC9/A496; Supplemental Figure S2A, http://links.lww.com/HC9/A496). These elements are responsive in both directions (some decrease in chromatin accessibility following stimulation while others increase), and enrichment is observed across all 5 T-cell lineages analyzed (Supplemental Figure S2B, http://links.lww.com/HC9/A496). Heritability is not significantly enriched for nonresponsive T-cell regulatory elements (Figure 3C).

Our aforementioned analysis of the immune cell panel (Figure 2B) also reveals PSC heritability enrichment in CD8^+^ T-cell regulatory elements responsive to stimulation. We asked whether this effect derives from a shared TCR response pathway between CD4^+^ and CD8^+^ lineages or separate TCR response pathways. We performed the same differential element analysis in public ATAC-seq datasets for 4 CD8^+^ T-cell lineages. While many TCR-responsive elements are shared between CD4^+^ and CD8^+^ T cells, several thousand are unique to each (Supplemental Figure S3, http://links.lww.com/HC9/A496). Heritability enrichment is strongest in the shared set (Figure 3D). Still, we also observe enrichment in the CD8^+^-specific set, implicating separate TCR response pathways in CD4^+^ and CD8^+^ T cells in PSC pathophysiology.

Taken together, these results suggest an underappreciated role for regulatory elements involved in response to TCR stimulation in the pathophysiology of PSC. Sixteen of the 29 non-HLA PSC-risk loci contain at least 1 TCR-responsive element (Supplemental Table S4, http://links.lww.com/HC9/A498; Figure 3E); some elements become more active following stimulation while others become less active, suggesting genetic disturbances could disrupt both positive and negative feedback loops during the TCR response.

### The epigenetic and transcriptomic landscapes of peripheral CD4^+^ T cells in PSC patients and controls

**FIGURE 4:**
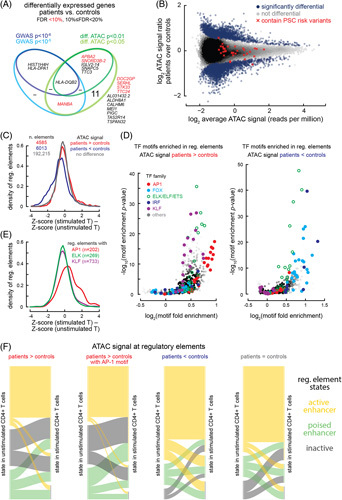
The epigenetic and transcriptomic landscapes of CD4^+^ T cells in PSC patients and healthy controls. A, A Venn diagram showing genes differentially expressed between patients and controls at 2 FDR significance thresholds (red and black gene symbols) which were also supported by differential ATAC-seq in patients versus controls (green ellipses) or PSC GWAS (blue ellipses). B, MA plot showing regulatory elements that had differential chromatin accessibility (blue) and nondifferential elements (gray) in patient versus control CD4^+^ T cells. Four PSC-risk loci (red X’s) contain variants that intersect a differentially accessible element. C, Histogram showing how many elements that had differentially accessible chromatin in PSC patients versus controls (blue and red curves) or nondifferential (gray curve) also exhibited differentially accessible chromatin following TCR stimulation in T cells based on public ATAC-seq data of asymptomatic donors.^14^ Positive *x*-axis values indicate increased chromatin accessibility following TCR stimulation; negative values indicate decreased accessibility on stimulation. D, Volcano plots showing TF motif enrichment within regulatory elements with more (left) or less (right) accessible chromatin in patients than controls. E, Histogram showing regulatory elements that had more accessible chromatin in PSC patients than healthy controls which contain one of 3 families of TF motifs; *x*-axis values indicate differential activity following TCR stimulation based on public ATAC-seq.^14^ Elements containing AP-1 motifs (red) have more accessible chromatin in stimulated T cells, while elements containing the other 2 motif families do not. F, Alluvial plots illustrating transitions in chromatin state for differential regulatory elements following TCR stimulation based on ENCODE data. Elements with more accessible chromatin in patients than controls (left 2 plots) are more likely to transition from inactive (gray) or poised enhancer (green) states to the active enhancer state (yellow) on stimulation, while elements with more accessible chromatin in controls (right center) or unchanged (right) are more likely to transition from active to inactive or poised states on stimulation. Abbreviations: ENCODE, Encyclopedia of DNA Elements; FDR, false discovery rate; LD, linkage disequilibrium; MA, Log Ratio vs Mean Average Plot; PSC, primary sclerosing cholangitis; TCR, T-cell receptor; TF, transcription factor.

Motivated by PSC’s heritability enrichment in T-cell regulatory elements, we profiled the epigenetic and transcriptomic landscapes of unstimulated peripheral CD4^+^ T cells in 10 PSC patients and 11 healthy controls using ATAC-seq and RNA-seq. A total of 125 genes are differentially expressed between patients and controls at a false discovery rate <10% and 250 at a false discovery rate <20%, and 1998 differentially accessible regulatory elements at *p*-value < 0.01 and 10,598 at *p*-value < 0.05 (Supplemental Table S5A, B, http://links.lww.com/HC9/A499). We aimed to link differential gene expression with differential regulatory elements and GWAS-supported PSC-risk SNPs using expressed quantitative trait loci (eQTLs) for the differential genes in CD4^+^ T cells from the DICE Project^37^ (Supplemental Methods, http://links.lww.com/HC9/A496). Twenty differential genes have either ATAC-seq or GWAS support, offering novel candidate risk genes for follow-up (Figure 4A). Two genes have both ATAC-seq and GWAS support; these include an HLA gene and *MANBA*, with the latter described in more detail below. Two additional GWAS-supported PSC-risk SNPs intersect a differentially accessible regulatory element (Figure 4B); these SNPs are eQTLs for *AMT* and *SIK2*, but these 2 PSC-implicated genes (Supplemental Table S1, http://links.lww.com/HC9/A494) are not differentially expressed between patients and controls. We also note an additional 13 candidate risk genes, 9 at the HLA locus and 4 not, for which an eQTL is a PSC GWAS-SNP (*p* < 10^−6^) and an eQTL falls within a differential regulatory element (*p* < 0.01) but the gene is not differentially expressed in our dataset (Supplemental Table S5C, http://links.lww.com/HC9/A499).

Given PSC’s heritability enrichment in TCR-responsive regulatory elements, we hypothesized that elements differentially accessible in patients versus controls might be TCR-responsive. Indeed, based on the public ATAC-seq data^14^ analyzed in Figure 3, elements less accessible in patients than controls become less accessible on TCR stimulation in healthy donors (blue line in Figure 4C; Kolmogorov-Smirnov test *p* = 3.1×10^−59^), while the elements more accessible in patients become slightly more accessible on TCR stimulation (red line in Figure 4C, *p* = 9.1×10^−6^). A possible interpretation is that PSC patients have a greater fraction of stimulated T cells circulating at baseline: the shape of the blue curve, in particular, suggests that regulatory elements which become silenced post-TCR stimulation (the negative *x*-axis portion of the blue curve in Figure 4C) are more likely to be silenced in patient cells.

We next evaluated whether any particular TFs modulate the differential elements, which might implicate particular pathways or immune cell lineages in PSC. We performed enrichment analysis using a comprehensive motif catalog we recently built from ENCODE TF ChIP-seq data.^38^ Regulatory elements more accessible in patients display a significant enrichment for the motifs of several TF families, including ELF/ELK/ETS factors, KLF factors, and AP-1 factors (Figure 4D, left panel). Elements less accessible in patients are enriched for ELF/ELK/ETS, FOX, and IRF motifs (Figure 4D, right panel).

AP-1 factors play key roles in TCR response pathways; TCR-responsive elements in Figure 3A display strong AP-1 enrichment (Supplemental Figure S4, http://links.lww.com/HC9/A496). Furthermore, AP-1 motif-containing elements increase accessibility following TCR stimulation in healthy donors (Figure 4E), while differential elements containing ELF/ELK/ETS motifs, KLF motifs, FOX motifs, and IRF motifs do not (curves for FOX and IRF omitted from Figure 4E for clarity). We used histone modification data from ENCODE to determine if these elements function as active enhancers in activated T cells (Supplemental Methods, http://links.lww.com/HC9/A496). Patient-more-accessible elements, particularly those containing the AP-1 motif, are more likely to transition from an inactive or poised enhancer state to an active enhancer state following TCR stimulation. In contrast, control-more-accessible elements are more likely to transition from an active enhancer state to a poised or inactive state following TCR stimulation (Figure 4F).

### The importance of assaying SNPs in the relevant immune cell type

**FIGURE 5:**
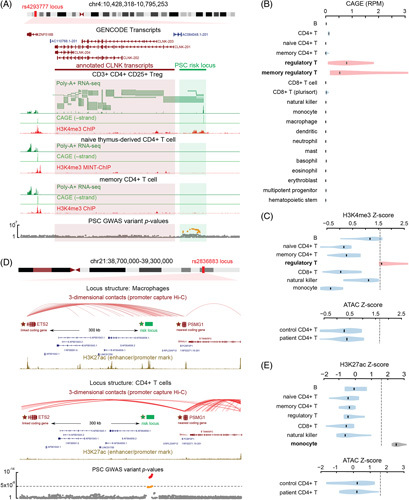
The activity of highly lineage-specific regulatory elements at 2 PSC-risk loci. A, Genome browser illustration of the 4q27 locus. Four transcripts for *CLNK* are annotated by GENCODE (maroon transcripts, center, corresponding to the pink-shaded region). ENCODE RNA-seq data (dark green tracks), FANTOM CAGE data (light green tracks), and ENCODE H3K4me3 MINT-ChIP data (red tracks) in 3 immune cell types highlight the existence of a candidate novel TSS for *CLNK* near PSC-risk variants (green shaded region) which is highly specific to Tregs. B, CAGE signal (reads per million; RPM) at the candidate novel TSS across a variety of immune cell types, illustrating Treg specificity. C, H3K4me3 signal at the locus in immune cells (top) illustrating specificity to Tregs; ATAC-seq signal (bottom) in PSC patient and control T cells indicating a lack of signal at the risk locus, likely due to the rarity of Tregs in peripheral CD4^+^ T cells. D, Genome browser illustration of the 21q22.2 locus. PSC-risk variants at the locus (green star, center) are 80 kbp from *PSMG1* and 300 kbp from *ETS2* (maroon stars and gene track). Enhancer activity is supported by a high H3K27ac signal in monocytes (top gold track) but not in CD4^+^ T cells (bottom gold track). Promoter-capture Hi-C data (red arcs) illustrate contact between the locus and the *ETS2* promoter in monocytes (top) but not T cells (bottom). E, H3K27ac signal at the 21q22.2 locus in various immune cells (top), illustrating monocyte specificity; ATAC-seq at the locus (bottom) in patient and control CD4^+^ T cells showing lack of chromatin accessibility. Abbreviations: *CLNK*, cytokine-dependent hematopoietic cell linker; ENCODE, Encyclopedia of DNA Elements; FDR, false discovery rate; GI, gastrointestinal; GWAS, genome-wide association studies; H3K4me3, trimethylation at histone H3 lysine 4; PSC, primary sclerosing cholangitis; Tregs, regulatory T cells; TSS, transcription start site.

There is potential regulatory element activity in peripheral CD4^+^ T cells from patients and controls at most PSC GWAS loci; however, a few elude characterization with our current dataset. Two examples are the 4p16.1 locus (lead SNP rs4293777) and the 21q22.2 locus (lead SNP rs2836883), neither of which has any PSC-risk variants within candidate regulatory elements in patient or control CD4^+^ T cells. Our analysis suggests a compelling reason for these negative results (Figure 5): PSC-risk variants at these loci intersect regulatory elements whose activity is highly specific to lineages of immune cells that we did not assay.

PSC-risk SNPs at the rs4293777 locus lie ∼60,000 base pairs upstream of *CLNK* (cytokine-dependent hematopoietic cell linker), encoding an adapter protein involved in immune receptor signaling. CAGE data from the FANTOM consortium^39^ suggest a novel transcription start site exists for *CLNK* used only in Tregs (Figure 5A, B). ENCODE RNA-seq in Tregs further supports this: paired-end reads span the unannotated transcription start site and annotated *CLNK* exons (Figure 5A). Trimethylation at histone H3 lysine 4, associated with active promoters, is observed at the locus in Tregs and no other immune cells, including other CD4^+^ T cells (Figure 5A, C). Thus PSC-risk variants at this locus may influence a novel promoter for a novel isoform of CLNK expressed only in Tregs. Because Tregs make up <5% of peripheral CD4^+^ T cells, it is not surprising that there is not sufficient signal to identify these effects in our current dataset (Figure 5C). Enriching and assaying Tregs will likely be essential to understand the pathophysiology of this locus.

There are 13 PSC-risk variants at the rs2836883 locus, which is associated with IBD^40^ in addition to PSC. The nearest coding gene, *PSMG1*, has been previously reported as a PSC-risk gene and is 80,000 base pairs away. Several variants intersect enhancers highly specific to monocytes based on acetylation of histone H3 lysine 27, characteristic of active enhancers (Figure 5D, E). Acetylation of histone H3 lysine 27 is not observed at the locus in other immune cell lineages, nor is chromatin accessibility in our patient or control CD4^+^ T cells (Figure 5E). Further, public promoter-capture Hi-C data illustrates 3D chromatin contacts specifically in monocytes between the PSC-risk locus and the promoter of a protein-coding gene, *ETS2*, ∼300,000 base pairs away (Figure 5D). *ETS2* codes for a proinflammatory TF whose binding sites are implicated in chronic inflammatory disease mouse models^41^ as well as human IBD.^42^ These results suggest that *ETS2* rather than *PSMG1* is the risk gene for PSC at this locus and suggest a role for monocytes in the pathophysiology of PSC. Follow-up studies in monocytes will be essential to elucidate this role.

### Allele-specific regulatory element activity at PSC-risk loci

**FIGURE 6:**
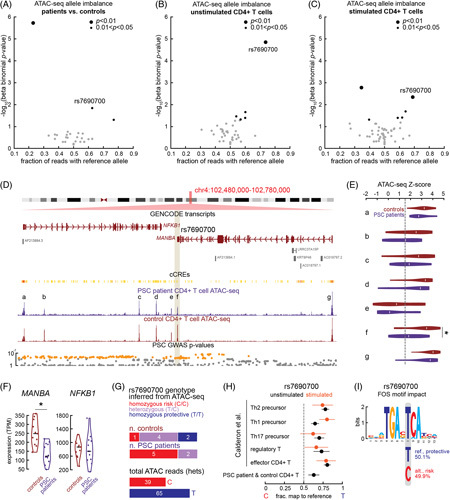
Allele-specific chromatin accessibility of T-cell regulatory elements at PSC-risk loci, highlighting the *MANBA* (4q24) locus. A–C, Allelic imbalances in the chromatin accessibility of regulatory elements containing PSC-risk variants in patients and controls (A), unstimulated T cells (B), and stimulated T cells (C) from public ATAC-seq data.^14^ The rs7690700 locus illustrated in Figure 5 replicates across all 3 datasets. D, Genome browser view of the 4q24 locus. PSC-risk variants fall within the vicinity of *NFKB1* and *MANBA* (gene track, top). ATAC-seq in peripheral CD4^+^ T cells from PSC patients (purple) and controls (maroon) illustrate 7 regulatory elements (a–g) with high ATAC signals at the locus. One (f, center) is more active in controls than patients. E, Violin plots illustrating activity at the 7 regulatory elements (a–g), highlighting a significant difference in activity between patients and controls at f, which contains risk variant rs7690700. F, Normalized expression of *MANBA* and *NFKB1* in CD4^+^ T cells from controls (maroon) and patients (purple) based on our RNA-seq data as analyzed with DESeq2. *MANBA* is more expressed in controls, while *NFKB1* is not differentially expressed. G, Top, inferred genotypes of 7 patients and 7 controls with sufficient read depth at rs7690700 (at least 5 reads) based on our ATAC-seq data; 5 of 7 patients have the risk C/C genotype, while 6 of 7 controls have at least one protective T allele. Bottom, the number of reads from the 6 heterozygotes containing the risk allele (C, red) or the protective allele (T, indigo). H, Deep sequencing allele imbalance at rs7690700 in stimulated (orange) and unstimulated (black) T cells from 5 different lineages, showing a preference for greater accessibility with the T allele across all datasets. I, An AP-1 motif at the locus; the risk C allele for rs7690700 is predicted to decrease AP-1 binding at the motif. Abbreviation: PSC, primary sclerosing cholangitis. *ATAC-seq: p=6.5×10^-3^.

The number of patients and controls in our study makes possible allele-specific analysis, which correlates genotype with regulatory element activity. If ATAC-seq reads are more likely to contain one allele than another allele for a particular variant, the more frequent allele is typically associated with greater regulatory element activity. We considered all credible PSC-risk SNPs at all 29 risk loci with at least 2 heterozygotes in our dataset as inferred from ATAC-seq (Supplemental Methods, http://links.lww.com/HC9/A496). A total of 42 significant PSC-risk SNPs met these criteria, and 3 of them exhibit significant allele-specific regulatory element activity (*p* < 0.05; Figure 6A; Supplemental Table S6A, http://links.lww.com/HC9/A500).

We supplemented these results with analysis on public CD4^+^ T-cell datasets.^14^ These complement ours: with fewer individuals, they are less likely to have heterozygotes for each variant, but with multiple lineages profiled, they have greater sequencing depth and thus greater power to detect allele-specific effects. They also have data from stimulated T cells, offering the potential to detect TCR response–specific differences. We identify significant allele-specific activity for 5 PSC-risk variants in unstimulated T cells and 6 in stimulated T cells. One present in both contexts, rs7690700, is also identified in our patient and control dataset with concordant directionality (Figure 6B, C; Supplemental Table S6B, C, http://links.lww.com/HC9/A500). We highlight its locus in the next section.

### Regulation of *MANBA* by a PSC-associated enhancer in CD4^+^ T cells

We highlight 1 PSC-risk locus exhibiting both differential enhancer activity and gene expression in patient versus control CD4^+^ T cells. Located on chromosome 4, the PSC-risk variants fall between *MANBA*—a gene involved in oligosaccharide catabolism—and *NFKB1*—a widely expressed pleiotropic TF (Figure 6D). Our ATAC-seq identifies 7 regulatory elements at this locus, including promoters for *NFKB1* and *MANBA* and 5 enhancers (Figure 6D, a–g). Of these, one enhancer (labeled f in Figure 6D), lying between the 3′-end of *MANBA* and the 5′-end of *NFKB1*, is significantly more accessible in controls than in patients (Figure 6E). Based on ENCODE annotations, this element is highly specific to CD4^+^ T cells.

We expected *NFKB1* might be the locus’ risk gene given a known role in inflammatory signaling cascades; however, it is not differentially expressed (Figure 6F). *MANBA*, however, is—controls express it at a significantly higher level than PSC patients (Figure 6F). Interestingly, the beta mannoside disaccharide the corresponding protein catabolizes is known to be antigenic. Individuals with the risk genotype may be less able to catabolize this antigen.

The differential enhancer at this locus contains a PSC-risk variant, rs7690700, for which 2 alleles, T and C, occur at roughly equal frequency in the European population. C confers risk for PSC while T is protective. Of 10 patients and 11 controls in our dataset, 7 and 7 had sufficient ATAC-seq read depth to infer a genotype. Five patients are homozygous for the risk allele and 2 are heterozygous; of the controls, 2 are homozygous for the protective allele, 4 are heterozygous, and 1 is homozygous for the risk allele (Figure 6G). In heterozygotes, ATAC-seq reads containing T occur at ∼1.5× the frequency of reads containing C (Figure 6G). The public ATAC-seq datasets we analyzed do not have as many unique donors as our patient and control dataset; nonetheless, they can provide valuable confirmatory evidence. Indeed, the same allelic imbalance pattern is observed in all CD4^+^ T-cell datasets from the Calderon dataset (Figure 6H).

We asked whether any TF binding motifs are disrupted by rs7690700 that might offer a mechanism for its impact. The protective T allele belongs to an AP-1 family motif within the enhancer; the risk C allele is expected to decrease the affinity of AP-1 factors for this motif (Figure 6I). A full hypothesis is that the risk allele decreases AP-1 binding at the enhancer in CD4^+^ T cells decreasing its activity; this, in turn, decreases the expression of MANBA and production of beta mannosidase lessening the ability to catabolize antigenic glycans.

## DISCUSSION

Based on our results, most PSC-risk loci have regulatory element activity, primarily in T cells. PSC heritability is not enriched in regulatory elements of GI or biliary epithelial cells; thus, our results suggest that a primary pathophysiological mechanism for noncoding PSC-risk variants is the disruption of immune-specific regulatory elements. We propose a role for TCR-responsive enhancers in PSC and illustrate 2 loci where variants may disrupt a lineage-specific promoter and enhancer in Tregs and monocytes, respectively. Studying these loci in the appropriate cell type and state will be essential to elucidate their pathophysiological role. The latter observation will represent a novel role for innate immunity in PSC if borne out.

Results from ATAC-seq and RNA-seq in PSC patients and healthy controls, while preliminary and with limitations described below, support and extend recent findings that peripheral T cells in PSC patients exhibit unique epigenetic signatures versus the general population.^43^ We present novel observations that AP-1 and KLF TF motifs are enriched in patient-preferred regulatory elements and FOX and IRF motifs are enriched in control-preferred elements. KLF4 and KLF10 regulate differentiation along the Th17/Treg^44,45^ axis, and FOXP3 is a key TF in Treg differentiation and function,^46^ possibly highlighting differences in polarization along the Th17/Treg axis in patient and control cells. AP-1 enrichment suggests patient cells are more stimulated, or primed to become stimulated, at baseline than those of controls.

We identified risk variants associated with differentially active CD4^+^ T-cell regulatory elements at 3 GWAS loci. We highlight rs7690700, which we propose dampens enhancer activity in patient T cells, thereby reducing expression of *MANBA*; we suggest this may reduce patients’ ability to catabolize antigenic glycans. Despite these observations, however, PSC-risk variants are not significantly enriched within differentially accessible regulatory elements in our dataset. Studying stimulated T cells will likely be an important follow-up. Because we studied unstimulated CD4^+^ T cells, regulatory elements accessible following TCR stimulation (orange bars in Figure 3C) are likely not captured. Elements which become less accessible following TCR stimulation (green bars in Figure 3C) should be; one possibility is that these elements would become silenced in control T cells following TCR stimulation but would remain inappropriately accessible in patient T cells. These elements would appear accessible but not differential in our TCR-unstimulated dataset.

Negative results at other GWAS loci may also owe in part to limitations in our study design. Our healthy control population was not strictly age-matched and sex-matched to the PSC patient population, and so these variables may confound the results to some degree. We did seek to minimize confounding comorbidities in both populations: none of the PSC patients or healthy controls were diagnosed with any other comorbid immune-mediated condition with the exception of IBD—all but 1 PSC patient was diagnosed with comorbid IBD, while no healthy controls were. In addition, none of the patients had advanced-stage PSC. More careful study design will be critical to limit such confounders in the follow-up study. In addition, inclusion of a comparison cohort with primary biliary cholangitis was beyond the scope of this study but may prove a fruitful avenue for elucidating features specific to PSC pathophysiology.

Overall, we offer preliminary results suggesting the pathophysiology of PSC includes genetic differences in gene regulation in 3 distinct immune processes. First, through major histocompatibility complex genotypes and possibly non–major histocompatibility complex genotypes, including the MANBA locus, patients may be more responsive toward antigens responsible for inciting the initial inflammatory response leading to PSC. Second, T cells from PSC patients may be genetically predisposed to respond more aggressively toward stimuli and to polarize away from Tregs, increasing the risk for a robust adaptive immune response. Third, possibly through genotype at the ETS2 locus, the monocytes of patients with PSC may be predisposed toward a proinflammatory phenotype, favoring the maintenance of a chronically proinflammatory biliary environment in established PSC.

In summary, the lineage specificity of the regulatory elements in our catalog introduces a novel framework for investigating the genetic and epigenetic factors underpinning the pathophysiology of PSC. By allowing for the prioritization of regulatory elements based on cell lineages and contexts, this framework can direct the design of translational studies that could advance our understanding of the pathophysiology, and ultimately the treatment, of PSC.

## Supplementary Material
