# Supplementary material for: Beyond genome-wide association studies: Investigating the role of noncoding regulatory elements in primary sclerosing cholangitis
Source: Hepatol Commun. 2023 Sep 27;7(10):e0242. doi: 10.1097/HC9.0000000000000242 (PMC10531193; doi:10.1097/HC9.0000000000000242)
Supplement: Supplementary file 3 [file hc9-7-e0242-s003.docx]

**Supplementary Figures**


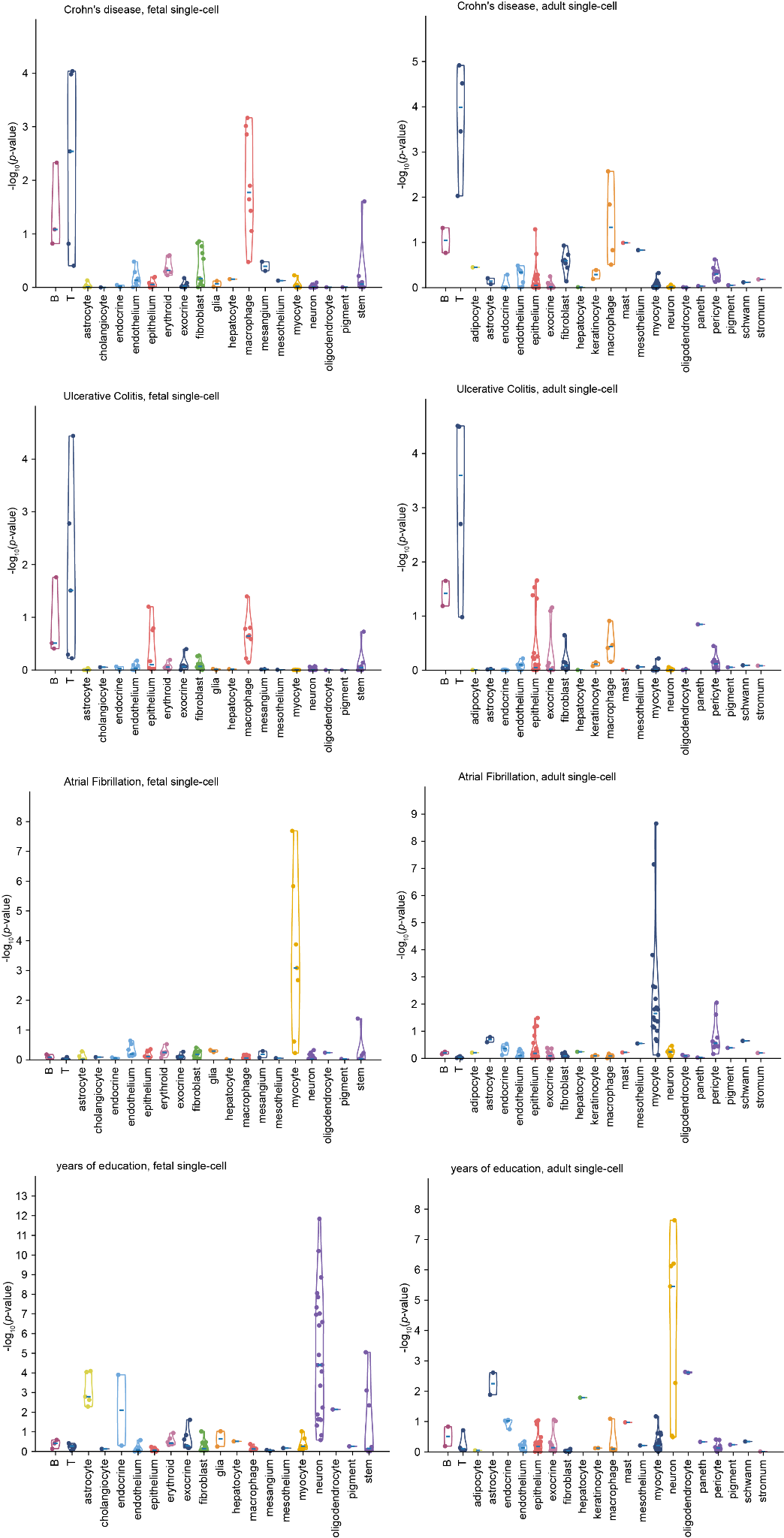


**Supplementary Fig. 1**. Heritability enrichment within single cell regulatory elements for a variety of fetal (left) and adult (right) cell types in Crohn’s disease (top row), ulcerative colitis (second row), atrial fibrillation (third row), and years of education (bottom row) as computed by partitioned LDSC.


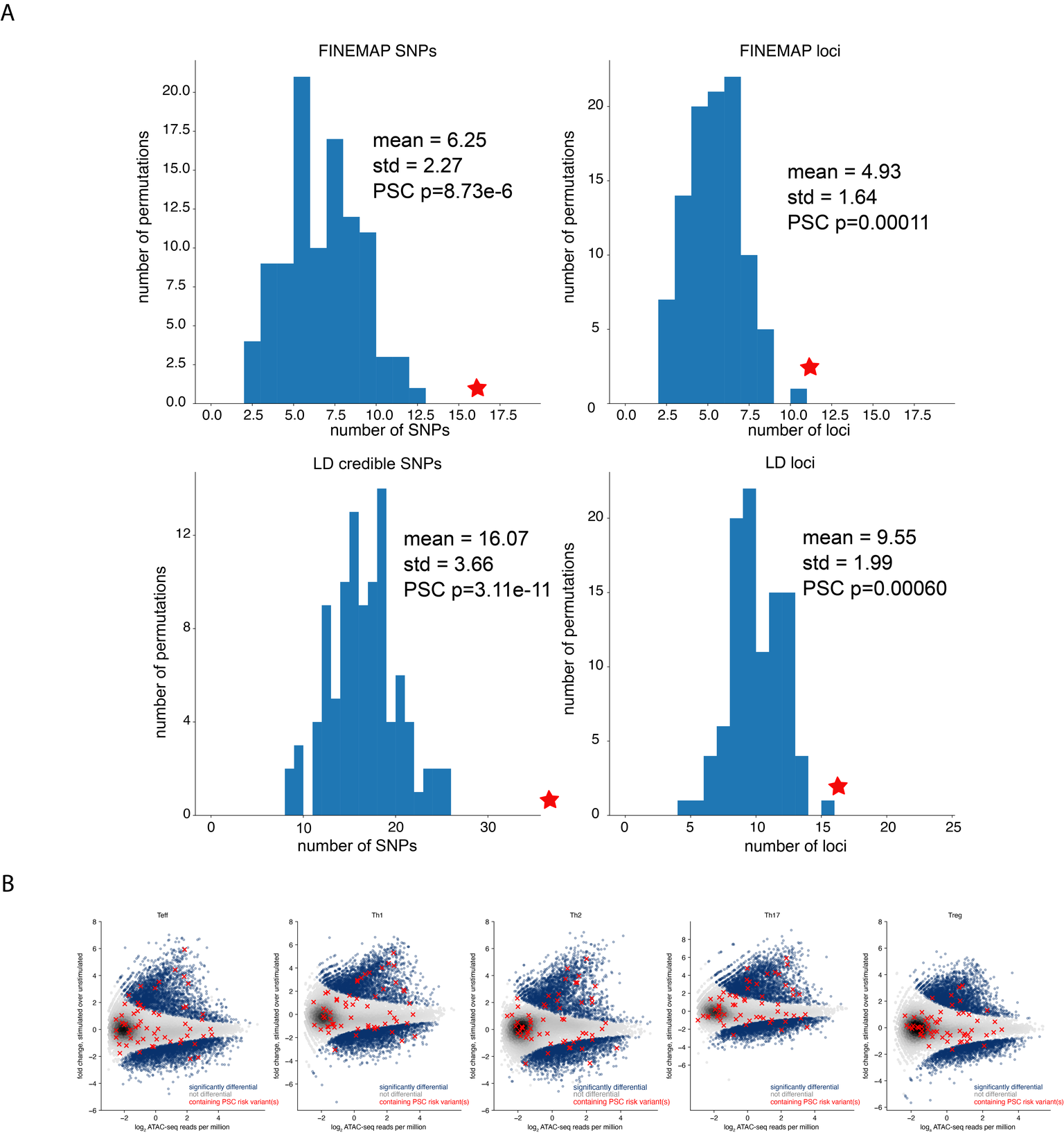


**Supplementary Fig. 2**. **A**, the number of credible PSC risk SNPs (left panels) or PSC risk loci (right panels) which intersect TCR-responsive regulatory elements (red stars) as compared with permuted negative control sets of variants and loci (histograms). **B**, MA plots illustrate significantly TCR-responsive T cell regulatory elements (blue) in five different CD4+ T cell lineages. Elements containing PSC risk variants are marked with red X’s.


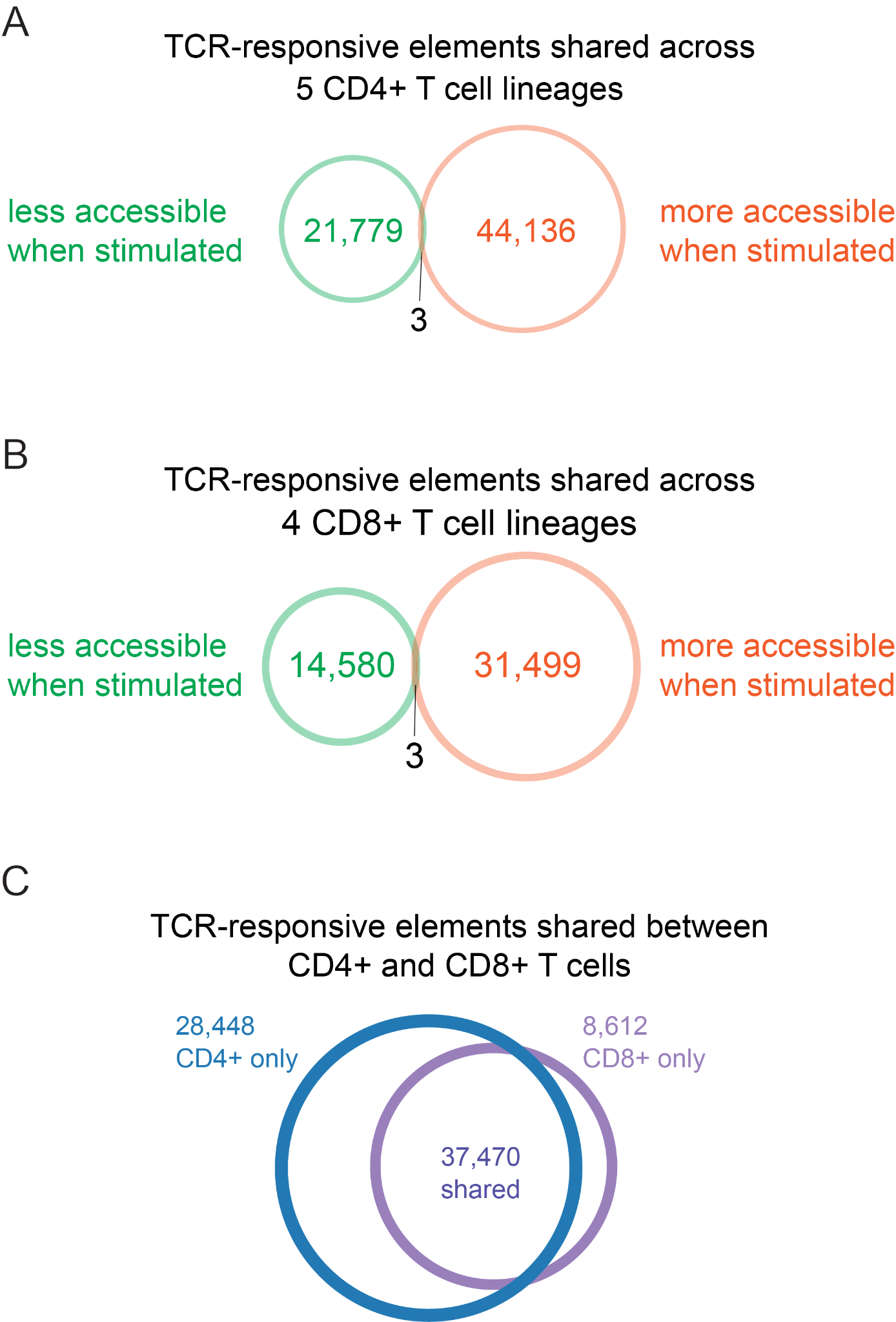


**Supplementary Fig. 3**. Venn diagrams indicating overlap between CD4+ and CD8+ TCR-responsive regulatory elements. **A-B**, the directionality of change in regulatory element accessibility is nearly perfectly preserved across CD4+ and CD8+ T cell lineages. **C**, illustration of shared and CD4+/CD8+-unique collections of TCR-responsive elements. A few thousand elements are TCR-responsive only in either CD8+ or CD4+ T cells.


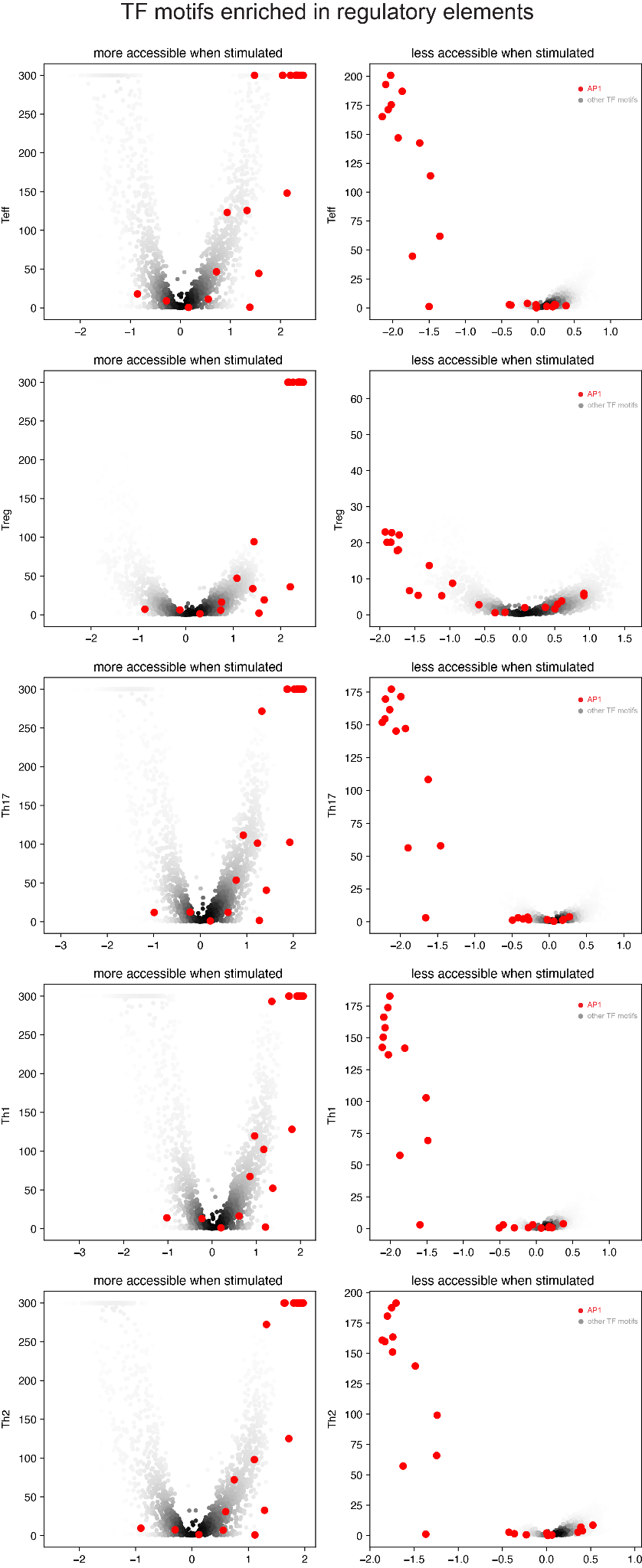


**Supplementary Fig. 4**. Volcano plots illustrate transcription factor motif enrichment within TCR-responsive elements which become more accessible (left) or more accessible (right) post-stimulation in five CD4+ T cell lineages. AP-1 motifs (red) are significantly enriched within elements which increase in accessibility and are significantly depleted within elements which become less accessible.

# **Supplementary Tables:**

#

# **Supplementary Table 1. Variants in the 29 non-HLA PSC-risk loci.**

1. Lead SNPs, notable genes, and credible SNP counts at each of the 29 non-HLA PSC-risk loci.
2. Credible PSC risk SNPs based on European linkage disequilibrium.
3. Credible PSC risk SNPs based on fine mapping with FINEMAP.
4. Deleterious coding annotations associated with credible risk variants from B and C.
5. List of all PSC risk loci annotated by whether credible variants at the locus intersect coding exons.
6. Overview of the regulatory element impacts of credible risk SNPs at the PSC risk loci.

#

# **Supplementary Table 2.**

1. LDSC results for PSC within regulatory elements from fetal bulk DNase-seq samples from ENCODE.
2. LDSC results for PSC within regulatory elements from immune primary cells and primary cholangiocytes.
3. LDSC results for PSC within regulatory elements from single cell ATAC-seq in adult tissues.
4. LDSC results for PSC within regulatory elements from single cell ATAC-seq in fetal tissues.
5. List of histone modification MINT ChIP datasets analyzed, with cell state information and unique accession numbers.

#

# **Supplementary Table 3.**

1. LDSC results for UC, AFib, and years of education for regulatory elements from single cell ATAC-seq in adult tissues.
2. LDSC results for UC, AFib, and years of education for regulatory elements from single cell ATAC-seq in fetal tissues.

#

# **Supplementary Table 4.**

1. List of credible SNPs based on LD which intersect regulatory elements which become more or less accessible following TCR stimulation.
2. List of credible SNPs based on fine mapping with FINEMAP which intersect regulatory elements which become more or less accessible following TCR stimulation.
3. List of credible SNPs based on LD and FINEMAP which intersect regulatory elements differentially accessible in CD4+ T cells from PSC patients vs. healthy controls.

**Supplementary Table 5.**

1. DESeq2 results showing differential expression of 250 genes in CD4+ T cells from PSC patients vs. healthy controls.
2. Regulatory elements differentially accessible in CD4+ T cells from PSC patients vs. healthy controls.
3. A list of genes which are (1) differentially expressed in patients vs. controls (from A), and/or (2) have at least one CD4+ T cell eQTL associated with PSC by GWAS, and/or (3) have at least one eQTL within a differential regulatory element from B.

**Supplementary Table 6.**

1. Allele specific analysis of SNPs within regulatory elements active in CD4+ T cells from PSC patients or healthy controls. Total read counts containing each allele from heterozygotes are shown, as well as allele imbalance *p*-value and whether or not the SNP is associated with one of the 29 non-HLA risk loci (significant) or associated with a locus of suggestive PSC association by GWAS (*p*<10^-6^; suggestive).
2. Allele specific analysis as in A, but for unstimulated CD4+ T cells from Calderon et. al.
3. Allele specific analysis as in A, but for stimulated CD4+ T cells from Calderon et. al.

**Supplementary Methods**

**PBMC isolation**

Peripheral blood mononuclear cells were isolated from 10 mL of blood donated by patients and healthy controls using Ficoll-Paque gradient separation. Whole blood was diluted 1:1 in phosphate-buffered saline (PBS) with 2 mM EDTA, layered onto Ficoll-Paque, and separated by centrifugation at 500g for 30 minutes. White cells from the buffy coat were extracted and washed twice in PBS with 2 mM EDTA with a 10-minute, 500g centrifugation after each wash. Cells were then resuspended in fetal bovine serum (FBS) with 10% dimethyl sulfoxide (DMSO) and brought to -80°C in a Mr. Frosty controlled-rate container for storage.

**CD4+ T cell isolation**

PBMCs isolated as per above were thawed rapidly. Untouched CD4+ T cells were then isolated using the MACS human CD4+ T cell isolation kit (Miltenyi Biotec catalog #130-091-155) per manufacturer instructions. The resulting T cells were then input directly into ATAC-seq and RNA-seq.

**ATAC-seq**

50,000 CD4+ T cells isolated as per above were used for ATAC-seq library building. The procedure was based on Kaestner lab’s ATAC-seq protocol: 50,000 cells were washed with PBS and resuspended with 50μl cold lysis buffer (10 mM Tris-HCl, pH 7.5, 10 mM NaCl, 3 mM MgCl 2 , 0.1% NP-40, 0.1% Tween-20, 0.01% digitonin). After 3 min incubation on ice, cells were washed with resuspension buffer (10 mM Tris-HCl, pH 7.5, 10 mM NaCl, 3 mM MgCl 2 , 0.1% Tween-20) and centrifuged at 500 g for 10 min at 4 °C. Supernatant was discarded, and pellet that contained nuclei was kept. For DNA tagmentation, nuclei were mixed with a 50 μl reaction mix that was composed of 25 μl Tagment DNA buffer (Illumina catalog #15027866), 16.5 μl PBS, 0.5 μl 10% Tween-20, 0.5 μl 1% digitonin, 2.5 μl TDE1 Tagment DNA enzyme (Illumina catalog #15027865), and 5 μl H2O. The reaction was incubated at 37 °C for 30 mins with shaking at 1,000 rpm. After tagmentation, DNA was purified using MinElute reaction cleanup kit (Qiagen catalog #28204) and eluted in 10 μl H2O. PCR amplification was carried out using NEBNext® high-fidelity 2X PCR master mix (NEB catalog #M0541S) and primers from IDT for Illumina DNA/RNA UD indexes set A tagmentation kit (Illumina catalog #20027213). 5 PCR cycles were applied, then a small portion of the partially amplified library was used to perform qPCR to determine the cycle number that effectively amplified the library but did not saturate the PCR reaction. The partially-amplified library was then continued with thermocycling with the determined cycle number. Some libraries were sequenced on an Illumina NextSeq 550 using NextSeq 500/550 high output kit v2.5 (Illumina catalog #20024906) with 75-base paired-end sequencing, and some were sequenced on an Illumina NovaSeq 6000 using 150-base paired-end sequencing.

**ATAC-seq uniform processing**

ATAC-seq deep sequencing reads from public datasets and our own experiments were processed using the ENCODE ATAC-seq uniform processing pipeline, described in detail at <https://www.encodeproject.org/atac-seq/> and available on GitHub at <https://github.com/ENCODE-DCC/atac-seq-pipeline>. Specifically, adapters were trimmed and reads were aligned to the hg38 reference genome using the Bowtie2 aligner, PCR duplicates were filtered using the Picard tool, and fold-change and *p*-value signal tracks were generated using MACS2.

Z-scores were generated for ENCODE representative DNase hypersensitive sites (rDHSs) using Version 3 of the ENCODE Registry of cCREs and ENCODE methodology [(1)](https://paperpile.com/c/IDA19Q/7AFyW). We computed Z-scores separately for all public ATAC-seq datasets and each of our own ATAC-seq datasets. In each dataset, the average *p*-value signal was computed for each rDHS, and then the mean and standard deviation of all non-zero signal means was computed. Z-scores were then computed for each rDHS by subtracting the overall signal mean from the rDHS’s signal value and then dividing by the overall signal standard deviation. rDHSs with zero signal were assigned a Z-score of -10. rDHSs were considered active in a given cell type using the ENCODE default threshold of Z>1.64.

**RNA-seq**

RNA extraction was performed on the CD4+ T cells from each sample which were not input to ATAC-seq using the RNA clean & concentrator-25 kit (Zymo Research catalog #11-353B). mRNA purification, first- and second-strand cDNA synthesis, 3’ adenylation, anchor ligation, and PCR amplification were performed using Illumina® stranded mRNA prep ligation 96 samples kit (Illumina catalog #20040534). Library clean-up was carried out by the Agencourt AMpure XP beads (Beckman Coulter catalog # A63881). Library indexing primers were from the IDT® for Illumina® RNA UD indexes set A ligation kit (Illumina catalog #20040553, 96 Indexes, 96 Samples). Details of the protocol can be found on the Illumina website. Libraries were sequenced on an Illumina NovaSeq 6000 with 150-base paired-end sequencing.

**RNA-seq uniform processing and differential expression analysis**

RNA-seq reads from our experiments were processed using the ENCODE RNA-seq uniform processing pipeline. Briefly, reads were aligned to the hg38 reference genome and transcriptome using STAR [(2)](https://paperpile.com/c/IDA19Q/sPeM4) with GENCODE v40 gene and transcript annotations. Gene and transcript quantifications were produced by RSEM. Differential expression analysis was performed using DESeq2 [(3)](https://paperpile.com/c/IDA19Q/Z2TKI) using raw read counts for each gene from the transcriptome BAM files produced by STAR using a custom Python script available in the code repository at <https://www.github.com/weng-lab/PSC-analysis/>.

**Histone mark analysis**

We downloaded fold-change signal files for MINT-ChIP of H3K27ac and H3K4me1 in stimulated and unstimulated effector CD4+ T cells from the ENCODE Portal (accession numbers in Supplementary Table 2e). We then computed the Z-scores of all ENCODE Version 3 rDHSs for each histone mark in each cell type using ENCODE methodology (rDHSs were expanded 500 base pairs on each side to capture the signal from flanking nucleosomes then analyzed as was ATAC-seq, described above). We define active enhancers in each cell state as all rDHSs having an H3K27ac Z-score >1.64 and poised enhancers as all rDHSs having an H3K4me1 Z-score >1.64 but an H3K27ac Z-score ≤1.64 in that cell state. rDHSs having Z-scores for both marks ≤1.64 were classed as inactive. We then performed an intersection between each type of enhancers against subsets of differential and non-differential regulatory elements from patient and control ATAC-seq data using bedtools.

**Motif enrichment analysis**

Motif enrichment was computed using rDHS motif instances from the Factorbook catalog. For each rDHS in the complete ENCODE set, we first generated a maximum Z-score across all PSC patients and healthy controls. We fit each rDHS set of interest, for example, elements more active in patients than controls, to a normal distribution of maximum Z-scores. We then drew ten random matched control sets of rDHSs from this distribution. Finally, we compared the number of instances of each Factorbook motif in the test set vs. the average number in the control sets using bedtools intersect followed by a Chi-square test to obtain an enrichment and *p*-value.

**PSC risk variant enrichment within TCR-responsive ATAC-seq sites**

We used the complete set of CD4+ T cell TCR-responsive elements described above, as well as the non-responsive set, to determine if PSC risk variants are enriched for intersection with TCR-responsive elements. We first intersected credible risk variants with the true CD4+ T cell differential set and counted how many total variants intersected. We next generated 100 random permutations of pseudo-TCR-responsive elements by (1) taking the union of responsive and non-responsive elements, then (2) randomly selecting a number of elements equal to the total number of true differential elements from the union set. We then intersected each of the random sets with the PSC risk variants and counted how many variants intersected in each instance. We computed a mean and standard deviation for variants intersecting the permuted sets and thus obtained a Z-score and *p*-value for the number of variants intersecting the true differential set.

1. [ENCODE Project Consortium, Moore JE, Purcaro MJ, Pratt HE, Epstein CB, Shoresh N, et al. Expanded encyclopaedias of DNA elements in the human and mouse genomes. Nature. 2020;583:699–710.](http://paperpile.com/b/IDA19Q/7AFyW)

2. [Dobin A, Davis CA, Schlesinger F, Drenkow J, Zaleski C, Jha S, et al. STAR: ultrafast universal RNA-seq aligner. Bioinformatics. 2013;29:15–21.](http://paperpile.com/b/IDA19Q/sPeM4)

3. [Love MI, Huber W, Anders S. Moderated estimation of fold change and dispersion for RNA-seq data with DESeq2. Genome Biol. 2014;15:550.](http://paperpile.com/b/IDA19Q/Z2TKI)
